# The efficacy of bilateral intervertebral foramen block for pain management in percutaneous endoscopic lumbar discectomy: A protocol for randomized controlled trial

**DOI:** 10.1097/MD.0000000000022693

**Published:** 2020-10-09

**Authors:** Xiaolan Sang, Hanmin Shan, Jiajun Hu, Meng Wu

**Affiliations:** a Department of operating room, Huzhou Central Hospital & Affiliated Central Hospital of Huzhou University; b Department of Anesthesiology; c Department of Orthopedics and Trauma, Huzhou Central Hospital & Affiliated Centre Hospital of Huzhou Unversity, Zhejiang Province, China.

**Keywords:** intervertebral foramen block, lumbar disc herniation, percutaneous endoscopic lumbar discectomy, protocol

## Abstract

**Background:**

Compared with open lumbar microdiscectomy, percutaneous endoscopic lumbar discectomy (PELD) has the advantages of remarkable preservation of paravertebral structures, less bleeding, shorter operation time and fewer complications, it is a common method for the treatment of lumbar disc herniation (LDH). Local anesthesia is recommended during PELD. However, intraoperative pain is sometimes difficult to control satisfactorily. The efficacy of bilateral intervertebral foramen block (IFB) for pain management in PELD remains unclear. Therefore, this regimen is utilized in a randomized controlled trial for the assessment the safety and effectiveness of bilateral IFB for PELD pain control.

**Method:**

This is a single center and randomized controlled trial which will be implemented from September 2020 to September 2021. This research protocol is in accordance with the items of the Standard Protocol for Randomized Trials, which was authorized through the Ethics Committee of Huzhou Central Hospital & Affiliated Centre Hospital of Huzhou University (HZCH0465-0864). 100 participants who undergo PELD will be analyzed. Inclusion criteria contains

1. patients diagnosed with LDH undergoing PELD;
2. people between the ages of 18 and 75;
3. consistent imaging evidence of herniation at a same level (CT or MRI);
4. receive conservative treatment for 2 months.

The exclusion criteria contains:

1. patients with the history of severe renal and hepatic dysfunction;
2. more than 1 responsible level
3. LDH combined with other spinal diseases needing advanced surgical treatment (such as fracture, spondylolisthesis, lumbar spinal stenosis, tumor, etc.);
4. Patients have mental illness that will prevent their willingness to participate in the study.

Patients will be randomly divided into bilateral IFB group (with 50 patients) and local infiltration analgesia group (with 50 patients). Primary outcomes are pain score at different time points. The secondary outcomes are the operative time, radiation exposure time, length of hospital stay and postoperative complications. All the analysis is implemented through applying the IBM SPSS Statistics for Windows, version 20 (IBM Corp., Armonk, NY, USA).

**Results:**

The clinical outcome variables between groups are illustrated in the Table 1.

**Conclusion:**

This investigation can offer a reliable basis for the effectiveness and safety of IFB in treating the PELD pain.

**Trial registration:**

This study protocol is registered in Research Registry (researchregistry5985).

## 1 Introduction

Lumbar disc herniation (LDH) is a familiar cause of lower extremity radiation pain and low back pain and conservative treatment can improve symptoms of most cases.^[1,2]^ About 80% of the patients suffered from sciatica caused by LDH, which imposes heavy burden on society, families, and individuals.^[3,4]^ In these cases, 10% to 20% of the patients received conservative treatment, but the pain continued, and surgery should be considered.^[5]^ Compared with open lumbar microdiscectomy, percutaneous endoscopic lumbar discectomy (PELD) has the advantages of remarkable preservation of paravertebral structures, less bleeding, shorter operation time, and fewer complications, it is a common method for the treatment of LDH.^[6,7]^

For patients with LDH undergoing surgery with spinal anesthesia or general anesthesia, it is hard to detect the accidental injury of the cauda equina nerve and nerve root due to sensory blockade.^[8]^ Although local anesthesia is recommended during PELD for avoiding nerve root injury, intraoperative pain is sometimes difficult to control satisfactorily under local anesthesia only,^[9,10]^ especially during the process of foraminoplasty, working channel insertion, and nucleus pulposus removal.

Severe pain during foraminoplasty and working channel insertion is considered to originate from the posterior longitudinal ligament. A local intervertebral foramen block (IFB) can block homolateral sinuvertebra nerve and spinal nerve located in the neuroforamen. Our preliminary research has indicated that IFB was associated with improved outcomes in terms of postoperative pain and opioid consumption. However, due to the small sample size and poor study design, the efficacy of bilateral IFB for pain management in PELD remains unclear. Therefore, this regimen is utilized in a randomized controlled trial for the assessment the safety and effectiveness of bilateral IFB for PELD pain control. We assume that that IFB is effective and safety in reducing postoperative pain in PELD.

## 2 Methods

### 2.1 Study design

This is a randomized controlled, single center trial which will be implemented from September 2020 to September 2021. This research protocol is in accordance with the items of the Standard Protocol for Randomized Trials, which was authorized through the Ethics Committee of Huzhou Central Hospital & Affiliated Centre Hospital of Huzhou University (HZCH0465–0864), and it has been registered in the research registry (researchregistry5985). All patients had signed consent forms before the surgery.

### 2.2 Population and randomization

100 participants who undergo PELD will be analyzed. In the random envelope, all patients are assigned a random number via using the random number (Table 1), and the result of allocation are hidden. Patients will be randomly divided into bilateral IFB group (with 50 patients) and local infiltration analgesia group (with 50 patients). Inclusion criteria contains

1. patients diagnosed with LDH undergoing PELD;
2. people between the ages of 18 and 75;
3. consistent imaging evidence of herniation at a same level (CT or MRI);
4. receive conservative treatment for 2 months.

**Table 1:**
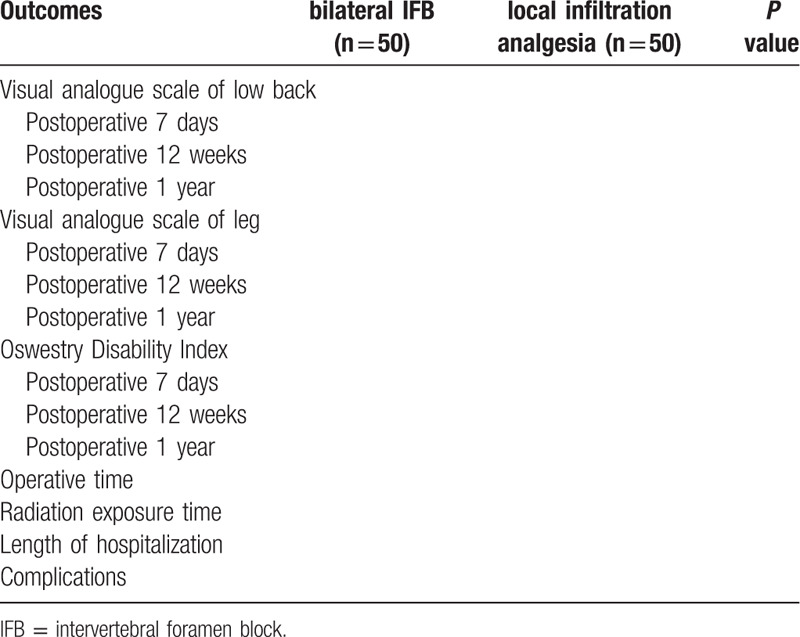
Comparison of follow-up outcomes among two groups.

The exclusion criteria contains:

1. patients with the history of severe renal and hepatic dysfunction;
2. more than one responsible level
3. LDH combined with other spinal diseases needing advanced surgical treatment (such as fracture, spondylolisthesis, lumbar spinal stenosis, tumor, etc.);
4. Patients have mental illness that will prevent their willingness to participate in the study.

### 2.3 Intervention

#### 2.3.1 Local anesthesia group

Patients receive the surgeries in prone position on the radiolucent Table 1. After 2 to 3 ml of 0.3% ropivacaine is infiltrated into the local skin, the selected skin entry point is pierced with an 18-gauge needle, and the track is anesthetized with 8 to 10 ml of 0.3% ropivacaine. The facet joint is anesthetized with 3 to 5 ml of 0.3% ropivacaine when the needle arrives at SAP. The following surgical procedures, such as removal of nucleus pulposus, insertion of working channel, and foraminoplasty, are the same as the procedures of conventional PELD.

#### 2.3.2 Bilateral IFB group

In case of lateral and anteroposterior fluoroscopy, 18-gauge is inserted into contralateral monosegmental intervertebral foramen. 2.5 ml of 0.3% ropivacaine is infiltrated into the contralateral intervertebral foramen for the free anesthesia. Subsequently, patients receive the traditional local anesthesia with 0.3% ropivacaine, which is the same as those of the local anesthesia group for SAP anesthesia, trajectory and homolateral skin. Then adjust the needle the homolateral monosegmental intervertebral foramen. A total of 2.5 ml of the 0.3% ropivacaine is infiltrated into the homolateral intervertebral foramen to conduct the sensory-motor dissociation anesthesia. The following surgical procedures is the same as the procedures of conventional PELD.

### 2.4 Outcome measures

Primary outcomes are pain score at different time point. Visual analog scale (VAS) is used to assess the pain (10: the maximum possible pain and 0: absent pain).^[11]^ The secondary outcomes are the operative time, radiation exposure time, length of hospital stay, and postoperative complications. The radiation exposure time is obtained from the G-arm computer at the end of each procedure. Oswestry Disability Index (ODI)^[12]^ is also recorded at 7 days, 12 weeks, and 1 year postoperatively.

### 2.5 Statistical analysis

All data are recorded into the Microsoft Excel 2010, and then they are analyzed via applying the IBM SPSS Statistics for Windows, version 20 (IBM Corp., Armonk, NY, USA). Afterwards, all the data are expressed with appropriate characteristics such as mean, median, standard deviation as well as percentage. Continuous and categorical variables are analyzed using χ^2^-tests and independent *t* tests, respectively. Intention-to-treat analysis is used for the outcome assessments. When *P* value <.05, it is considered to be significant in statistics.

## 3 Results

The clinical outcome variables between groups are shown in Table 1.

## 4 Discussion

To the best of our knowledge, this is the first randomized controlled trial to assess the safety and effectiveness of bilateral IFB for PELD pain control. LDH is a common orthopedic disease and a worldwide health problem characterized by low-back and radiating pain.^[13,14]^ PTED has become one of the optimal clinical treatments for LDH due to the advantages of minimal invasion, less blood loss, and rapid recovery.^[15,16]^ Surgery and anesthesia are associated with a stress reaction, immunosuppression, and postoperative pain, which prolong hospital stays and increase the economic burden of patients.^[9]^ Appropriate anesthesia is important to achieve improved clinical outcomes.^[17]^ The lack of a definitive “gold standard” and various programs of anesthesia method during operation indicate that there is much room for improving the perioperative pain control. Recently, IFB is popular used in PELD. However, if conducted unilateral IFB only, satisfactory and instant distribution of local anesthetic to the opposite side usually could not be obtained, due to the mass effect of herniated nucleus pulposus, spinal stenosis, intraspinal adhesion, different puncture site and other factors. Therefore, bilateral IFB is recommend for intraoperative pain management in PELD. Considering that the sample size is small. Our findings must be replicated in more research centers and in larger samples, and evaluate over longer periods of follow-up, before final conclusions can be drawn.

## 5 Conclusion

This investigation can offer a reliable basis for the effectiveness and safety of IFB in treating the PELD pain.

## Author contributions

Meng Wu designed and reviewed protocol. Hanmin Shan and Jiajun Hu performed the data collection and analysis. Xiaolan Sang finished the manuscript. All of the authors approved the submission.

**Conceptualization:** Hanmin Shan.

**Funding acquisition:** Meng Wu.

**Methodology:** Jiajun Hu.

**Writing – original draft:** Xiaolan Sang.

## Abbreviations

Abbreviations: IFB = intervertebral foramen block, LDH = lumbar disc herniation, ODI = Oswestry Disability Index, PELD = Percutaneous endoscopic lumbar discectomy.

## References

[1] Arts MP Kursumovic A Miller LE Wolfs J. Comparison of treatments for lumbar disc herniation: systematic review with network meta-analysis. Medicine (Baltimore) 2019;98 (7):e14410. 30762743 10.1097/MD.0000000000014410 PMC6408089

[2] Shepard N Cho W. Recurrent lumbar disc herniation: a review. Global Spine J 2019;9 (2):202–9. 30984501 10.1177/2192568217745063 PMC6448208

[3] Chen BL Guo JB Zhang HW Zhang YJ Zhu Y. Surgical versus non-operative treatment for lumbar disc herniation: a systematic review and meta-analysis. Clin Rehabil 2018;32 (2):146–60. 28715939 10.1177/0269215517719952

[4] Ilyas H Savage J. Lumbar disk herniation and sport: a review of the literature. Clin Spine Surg 2018;31 (9):366–72. 30045110 10.1097/BSD.0000000000000696

[5] Selva-Sevilla C Ferrara P Geronimo-Pardo M. Cost-utility analysis for recurrent lumbar disc herniation: conservative treatment versus discectomy versus discectomy with fusion. Clin Spine Surg 2019;32 (5):E228–34. 30839420 10.1097/BSD.0000000000000797

[6] Li X Han Y Di Z. Percutaneous endoscopic lumbar discectomy for lumbar disc herniation. J Clin Neurosci 2016;33:19–27. 27475315 10.1016/j.jocn.2016.01.043

[7] Peng CW Yeo W Tan SB. Percutaneous endoscopic lumbar discectomy: clinical and quality of life outcomes with a minimum 2 year follow-up. J Orthop Surg Res 2009;4:20. 19555483 10.1186/1749-799X-4-20 PMC2712454

[8] Sharma N Piazza M Marcotte PJ. Implications of anesthetic approach, spinal versus general, for the treatment of spinal disc herniation. J Neurosurg Spine 2018;30 (1):78–82. 30497221 10.3171/2018.7.SPINE18460

[9] Guan Y Huang T An G. Percutaneous endoscopic interlaminar lumbar discectomy with local anesthesia for L5-S1 disc herniation: a feasibility study. Pain Physician 2019;22 (6):E649–54. 31775418

[10] Wang SJ Chen BH Wang P. The effect of percutaneous endoscopic lumbar discectomy under different anesthesia on pain and immunity of patients with prolapse of lumbar intervertebral disc. Eur Rev Med Pharmacol Sci 2017;21 (12):2793–9. 28682439

[11] Faiz KW. VAS--visual analog scale. Tidsskr Nor Laegeforen 2014;134 (3):323. 24518484 10.4045/tidsskr.13.1145

[12] Zigler JE Delamarter RB. Oswestry disability index. J Neurosurg Spine 2014;20 (2):241–2. 24645201

[13] Yang H Liu H Li Z. Low back pain associated with lumbar disc herniation: role of moderately degenerative disc and annulus fibrous tears. Int J Clin Exp Med 2015;8 (2):1634–44. 25932092 PMC4402739

[14] Samini F Gharedaghi M Khajavi M. The etiologies of low back pain in patients with lumbar disk herniation. Iran Red Crescent Med J 2014;16 (10):e15670. 25763198 10.5812/ircmj.15670 PMC4329753

[15] Pan M Li Q Li S. Percutaneous endoscopic lumbar discectomy: indications and complications. Pain Physician 2020;23 (1):49–56. 32013278

[16] Kim M Lee S Kim HS. A Comparison of percutaneous endoscopic lumbar discectomy and open lumbar microdiscectomy for lumbar disc herniation in the korean: a meta-analysis. Biomed Res Int 2018;2018:9073460. 30175149 10.1155/2018/9073460 PMC6106715

[17] Buvanendran A Thillainathan V. Preoperative and postoperative anesthetic and analgesic techniques for minimally invasive surgery of the spine. Spine (Phila Pa 1976) 2010;35: (26 Suppl): S274–80. 21160390 10.1097/BRS.0b013e31820240f8

